# Appetite and Gut Hormones Response to a Putative α-Glucosidase Inhibitor, *Salacia Chinensis*, in Overweight/Obese Adults: A Double Blind Randomized Controlled Trial

**DOI:** 10.3390/nu9080869

**Published:** 2017-08-12

**Authors:** Lihong Hao, Yvette Schlussel, Krista Fieselmann, Stephen H. Schneider, Sue A. Shapses

**Affiliations:** 1 Department of Nutritional Sciences, Rutgers University, New Brunswick, NJ 08901, USA; lilyhlh@gmail.com (L.H.); schlusse@rutgers.edu (Y.S.); kfieselmann@gmail.com (K.F.); 2 The Rutgers Center for Lipid Research, New Jersey Institute for Food, Nutrition, and Health, Rutgers University, New Brunswick, NJ 08901, USA; 3 Department of Medicine, Division of Endocrinology, Nutrition and Metabolism, Rutgers-Robert Wood Johnson University Hospital, New Brunswick, NJ 08901, USA; steve.schneider@gmail.com

**Keywords:** appetite, glycemic indices, gastrointestinal peptides, *Salacia Chinensis*

## Abstract

Animal studies indicate *Salacia* reduces body weight, possibly due to its α-glucosidase inhibitor (α-GI) properties, but this has not been examined previously. In this study, a randomized, placebo-controlled, three-way cross-over design was used to evaluate whether *Salacia Chinensis* (SC) reduces appetite in healthy overweight/obese individuals (body mass index 28.8 ±3.6 kg/m^2^; 32 ± 12 years). Forty-eight participants were fasted overnight and consumed a dose of SC (300 or 500 mg) or placebo with a fixed breakfast meal at each visit. Appetite sensations, glycemic indices and gastrointestinal peptides were measured. Results indicated that SC had no effect on postprandial appetite. However, in women, hunger was reduced by SC compared to placebo at multiple time points (300 mg; *p* < 0.05), but not in men. Area under the curve (AUC) for serum glucose, insulin and amylin was attenuated with SC compared to placebo (*p* < 0.05). Glucagon like peptide-1 had two peaks after the meal, but the AUC did not differ between groups. The AUC of peak areas for peptide YY and ghrelin were greater for SC than placebo (*p* < 0.05). These findings indicate that *Salacia* decreases glycemic indices supporting its role as an α-GI, and affects certain gastrointestinal peptides suggesting it may be an appetite modulator.

## 1. Introduction

Obesity is a national health problem that has reached epidemic proportions. The prevalence of obese and overweight individuals in the US is 34% and 68%, respectively, and this population is at high risk of developing obesity-related morbidities, such as diabetes mellitus, hypertension, coronary heart disease and hyperlipidemia [1]. While nearly half the population is on a diet at any given time among those who lose weight, there is an approximately 80% recidivism rate to obesity among those formerly obese [2]. Both appetite and its regulation by gut peptides are important regulators of food intake, and an enhanced appetite after weight loss is at least one cause of weight regain [3,4,5,6,7]. Hence, the inability to be satiated can lead to excessive overeating in the obese both before, during and after weight loss.

Many animal and clinical studies have shown that α-glucosidase inhibitors (α-GIs), such as miglitol, acarbose and vodagliptin, have antidiabetic effects resulting in decreased blood glucose and insulin levels. In addition, α-GIs also have been found to affect appetite through enhancing satiety and suppressing hunger [8,9,10,11,12] that may explain their modest effect on weight loss [13]. Extracts of *Salacia*, a herbal plant that has α-GI properties, have been used in traditional Asian medicine for therapeutic and/or preventative treatment of various diseases, especially obesity and insulin resistance [14]. Studies have shown that *Salacia* acts as an α-GI to affect glucose metabolism, and that various species of *Salacia*, including *Salacia Chinensis* (SC), reduce postprandial plasma glucose and insulin in healthy subjects [15]. In addition, *Salacia* has been shown to reduce body weight in animal studies [16]. However, the mechanism inducing weight loss with *Salacia* has not been addressed. Thus far, there are no studies examining whether this putative α-GI alters appetite. The primary aim in this study was to determine whether SC affects hunger in healthy overweight/obese individuals after a mixed meal tolerance test. In addition, the effect of SC on glycemic indices, gastrointestinal hormones and taste sensations were also examined.

## 2. Materials and Methods

### 2.1. Participants

Healthy men and women who were overweight or obese with a body mass index (BMI) between 25–35 kg/m^2^ were recruited. All participants were recruited at the Department of Nutritional Sciences, Rutgers University through local newspapers and electronic advertisements (Facebook, etc.), and through email list serves every few months between 2015 and 2016. Study screening was recorded using Qualtrics (www.qualtrics.com; Qualtrics, Provo, UT, USA). Individuals were excluded if there was a diagnosis of an eating disorder, gastrointestinal illness, bariatric surgery, hyperparathyroidism, untreated thyroid disease, diabetes, blood pressure > 140/90, significant immune, hepatic, or renal disease, significant cardiac disease, active malignancy or cancer therapy within the past year, current use of obesity medications or dietary supplements or any weight regimen. Telephone interviews were conducted after participants filled out the screening form by trained staff to further determine whether subjects met the experimental criteria. Eligible volunteers underwent biochemical and physical screening including a comprehensive chemistry panel, complete blood count, and brief physical examination to ensure they were healthy and had no evidence of undiagnosed diseases (i.e., anemia, diabetes, high blood pressure). The study was approved by the Rutgers University Institutional Review Board and all study participants signed an informed consent form before entering the study. The trial is registered at clinicaltrials.gov (NCT02929849). The protocol met the ethical standards in accordance with the Helsinki Declaration.

### 2.2. Study Design and Procedure

**Figure 1:**
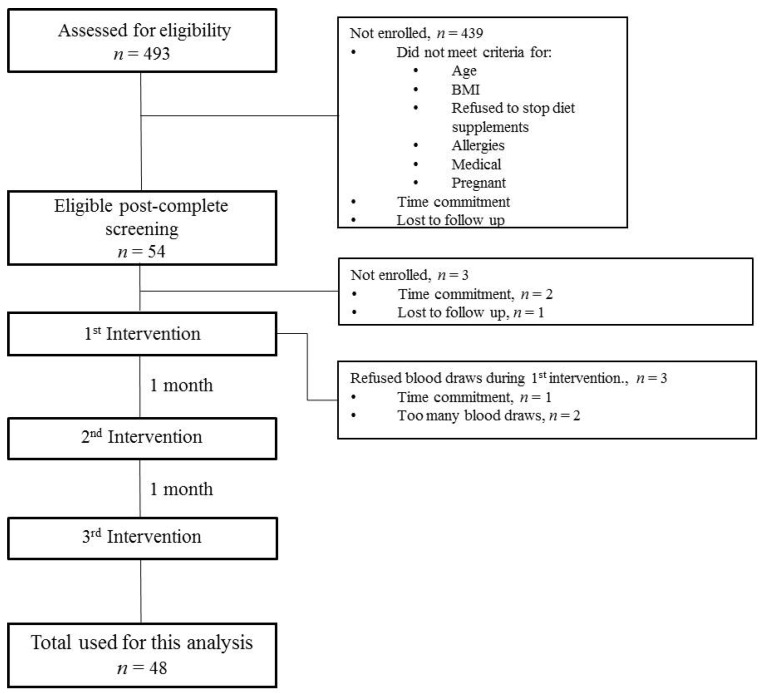
Flowchart of study participants. At each intervention, subjects were randomized in a double blind manner to placebo or one of two doses of treatment in a cross-over design.

In this double-blind, placebo-controlled, three-way cross-over trial, participants (aged 21–59 years) were randomly assigned to one of two doses of SC extract (300 mg or 500 mg) or placebo (Figure 1). There was a 1 month washout period between treatment visits to minimize the potential effect of varying concentrations of circulating estradiol that would be expected over the course of the menstrual cycle on the satiety outcomes. Participants were encouraged to consume the same dinner before each of the three test days. After an overnight fast (no intake after 9 pm), participants were asked to fill out a 24 h diet recall; had body weight, blood pressure, body fat, and waist circumstance measured; and had a baseline fasting (0 min) blood sample drawn. Participants also completed multiple visual analogue scales (VAS) before breakfast to measure their subjective appetite sensations (hunger, fullness, prospective food consumption and satiety) and taste perceptions (sweet, salty, savory and fatty). After baseline blood draw and VAS evaluation, each participant was served a breakfast meal (275 kcal; 50% carbohydrate; 30% fat; 20% protein), and a capsule containing color and size matched placebo or one of the two doses of SC (OmniActive Health Technologies Ltd., Morristown, NJ, USA) in 10 min. Blood samples and VAS evaluation were obtained at six time points before and during the 3 h postprandial period (0, 30, 60, 90, 120 and 180 min) on each of the 3 days of measurement.

### 2.3. Measurements

#### 2.3.1. Appetite Measures

The VAS was used to assess appetite (hunger, fullness, prospective food consumption and satiety), and subjective taste perceptions (sweet, salty, savory and fatty). The scale has a 100 mm horizontal line with the most positive and the most negative rating at each end. Participants chose a point on the line that would be representative of their current perception for satiety. The distance in mm between the leftmost (zero) point and the point marked by participant was measured and used to determine the VAS score. Participants used a Kindle reader (Amazon.com) for the VAS self-assessment. Participants were not allowed to socialize or discuss their ratings during the test or see their previous VAS questionnaire recordings [17].

#### 2.3.2. Serum Biochemistry

Six blood samples on the 3 study days were collected in serum-separating tubes and allowed to clot at room temperature. Tubes were centrifuged for 1000× *g* for 15 min at 4 °C, and protease inhibitor (Aprotonin, Sigma-Aldrich, St. Louis, MO, USA) was added into the serum. Samples were stored at −80 °C before batch analysis at the end of the trial. Serum glucose was measured using YSI 2300 STAT PLUS Glucose Analyzer (YSI Incorporated, Yellow Springs, OH, USA). Serum insulin was measured with commercially available enzyme linked immunoassay kits (ELISA, MybioSource.com). Serum ghrelin was measured using human ELISA assay kits (EMD Millipore, Billerica, MA, USA; inter- and intra- assay % coefficient of variations (CVs) were 6.2%–7.8% and 0.9%–1.3%, respectively). Serum peptide YY (PYY), amylin and glucagon-like peptide-1 (GLP-1) were measured using a Multiplex magnetic-bead based immunoassay kits (EMD Millipore, Billerica, MA, USA). The inter-assay CV was <20% for amylin and <15% for all others. All assays had an intra-assay CV < 10%.

### 2.4. Sample Size Estimation

Power calculations were made according to previously validated methods [17,18]. Our goal was to assess outcomes of either treatment (300 mg SC or 500 mg SC) compared to placebo. Twenty subjects per group would allow us to detect a 10% difference in the primary appetite rating score outcome, hunger, compared to placebo (80% power and *p* value < 0.05). This sample should have also been adequate to detect differences in other appetite scores (fullness, satiety and prospective food intake). If there is a 30 area under the curve (AUC) difference between groups during the post-prandial period with a standard deviation (SD) of 22 and power of 90%, 11 subjects per group would be needed to detect a difference of treatment compared to placebo, as examined previously with another appetite suppressant food supplement vs. placebo [18]. In addition, a power analysis for a difference (*Salacia* vs. placebo) in positive AUC and peak concentration for glucose or insulin indicated that we needed 6 and 16 subjects, respectively at 90% power [19]. The order of treatment was randomly assigned using SPSS randomization program (version 24, IBM Corp, Armonk, NY, USA), and both participants and clinical staff were blinded to the treatment.

### 2.5. Statistical Analysis

Descriptive statistics of survey respondents and survey results were calculated. Chi-square test was used to examine the relationship between demographic variables. Normality of the data was tested using Shapiro-Wilk′s test of normality. All data obtained in this study were normally distributed. For missing data, last value carried forward was used for the intention to treat (ITT) analysis. Within and between-group comparisons of means from before and at each postprandial time point (0, 30, 60, 90, 120, and 180 min) were performed using a general linear model for repeated measures ANOVA with Bonferroni correction. In addition, individual values were adjusted for baseline and examined for change at each time point if there were baseline differences between treatment days. The area under the curve (AUC) for peak areas of blood variables was calculated [19,20]. The variability in response by sex (male vs. female) and BMI category (overweight vs. obese) on outcomes were also examined. Between-group comparisons were conducted using one-way ANOVA. We also compared treatment at each time point vs. placebo (treatment minus placebo values) by a one sample t-test at each time point. Pearson correlations were used to determine relationships between appetite and gut hormones. Additional statistical analysis was examined using linear models of two-way interactions between main effects (SC treatment) and order of treatments. All statistical analyses were performed using an intention to treat analysis using SPSS statistical software (IBM, version 24.0). Data are represented as mean ± SD, unless otherwise indicated. A *p* value < 0.05 was considered statistically significant.

## 3. Results

A total of 493 participants were screened and 54 participants were eligible for recruitment by meeting the inclusion/exclusion criteria; 54 participants enrolled and 48 successfully completed the mixed-meal tolerance test, participating in all three protocol days. For the 6 participants who were not included in the study, 3 withdrew before any treatment visits, and 3 withdrew after the first treatment visit (Figure 1). The characteristics of 48 participants are shown in Table 1.

Individuals were from a diverse racial background (43% Caucasian, 33% Asian, 15% African American, 7% Hispanic). There were 28 females (60%) and 20 males (40%), and BMI indicated that there were 29 overweight (26.2 ± 1.6 kg/m^2^) and 19 obese (32.7 ± 1.9 kg/m^2^) individuals (Table 1). Serum biochemical values represent fasting morning values (mean of three days), and no values differed significantly between visits. Twelve (25%) of 48 participants had a fasting screening glucose concentration above 100 mg/dL that averaged 104.8 ± 5.3 mg/dL. In addition, the homeostatic model assessment-insulin resistance (HOMA-IR) and quantitative insulin sensitivity check index (QUICKI) were 3.8 ± 2.0 and 0.32 ± 0.02, respectively.

### 3.1. Appetite Measures

**Figure 2:**
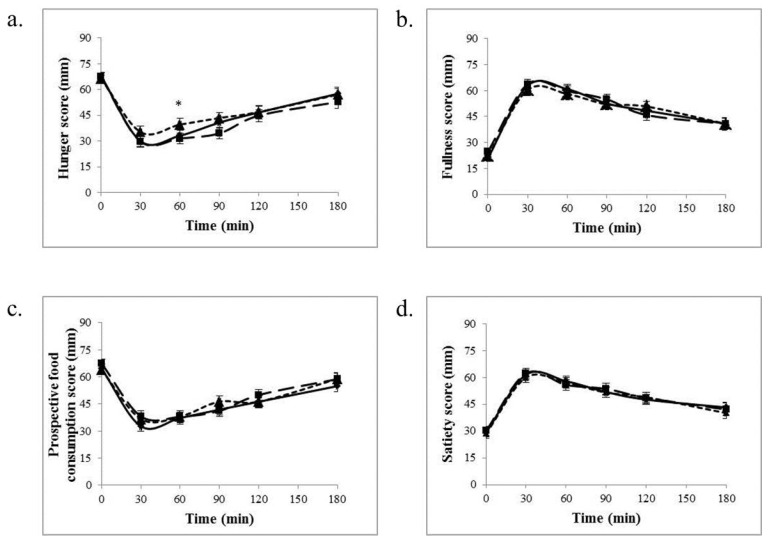
Visual analogue scales (VAS) scores for hunger (**a**), fullness (**b**), satiety (**c**), and prospective food consumption (**d**) during 3 h after the mixed breakfast meal with a dose of *Salacia Chinensis* (SC) or placebo (*n* = 48). * *p* < 0.05, Differs compared to placebo. Values are means ± standard error of the mean (SEM). Placebo: 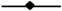; 300 mg: 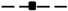; 500 mg: 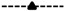.

There was no significant effect of treatment for hunger, fullness, satiety and prospective food consumption using repeated measures ANOVA. Analysis of postprandial hunger indicated it was greater at one time point in the SC group (500 mg) compared to placebo (*p* < 0.05) (Figure 2). Postprandial integrated AUC did not differ between groups for any appetite measures. No significant effect of treatment for taste perceptions was observed when compared to placebo (data not shown).

**Figure 3:**
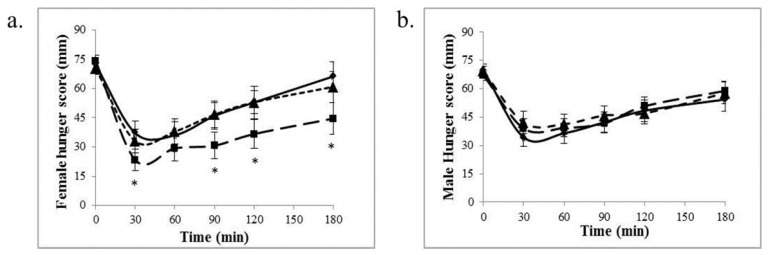
VAS hunger scores during 3 h in age-matched females (**a**) and males (**b**). *n* = 32. * *p* < 0.05, Differs compared to placebo. Values are mean ± SEM. Placebo: 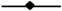; 300 mg: 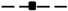; 500 mg: 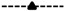.

In a multiple regression analysis using age, sex and BMI in the model to examine appetite measures, only sex significantly predicted postprandial hunger response (beta = 0.22; *p* < 0.02) at 30 min. There were no predictors of any of the other appetite measures. When measurements were examined in women, postprandial hunger scores were lower with SC (300 mg) than placebo at 90 and 180 min (*p* < 0.05). Because the mean age and range of ages in women was greater than men, we examined hunger scores in an age-matched group (< median of 34 years) and found that the lower hunger scores in younger women remained significant, and at more time points (30, 60, 90 and 180 min; *p* < 0.05) (Figure 3a).

### 3.2. Serum Glycemic Indices and Gut Peptides

There was no significant difference in fasting concentrations of serum glucose, insulin, amylin, GLP-1, PYY and ghrelin between the three study days and mean values are shown in Table 1. PYY concentrations are reported for 33 subjects that had detectable levels.

**Figure 4:**
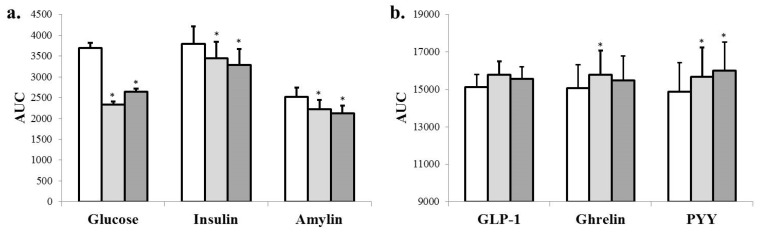
Integrated area under the curve (AUC) for peak areas of glycemic indices (**a**) and gut hormones (**b**) after a mixed meal with either SC treatment compared to placebo. The AUC units are as follows: glucose (mg × min/dL); insulin (µIU × min/mL); and amylin/GLP-1/ PYY (pg × min/mL). * *p* < 0.05, Differs compared to placebo for glucose (×5), insulin, amylin, ghrelin (×0.5) and PYY. Placebo: 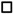; 300 mg: 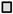; 500 mg: 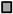.

**Figure A1:**
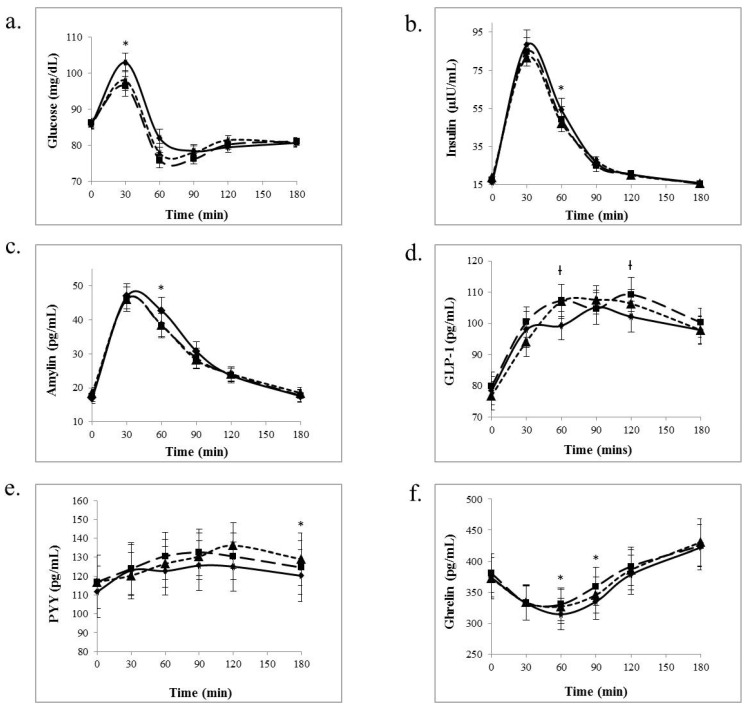
Serum glucose (**a**), insulin (**b**), amylin (**c**), GLP-1 (**d**), PYY (**e**) and ghrelin (**f**) levels during 3 h after the mixed breakfast meal with a dose of SC or placebo. Abbreviations: glucagon-like peptide-1 (GLP-1), peptide YY (PYY), *Salacia Chinensis* extract (SC). * *p* < 0.05; ƚ *p* < 0.06, Differs from placebo. Values are means ± SEM. Placebo: 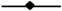; 300 mg: 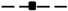; 500 mg: 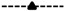.

After the mixed meal, the AUC for peak areas of glucose, insulin and amylin was attenuated by each SC treatment compared to placebo (Figure 4a, *p* < 0.05). The meal tolerance curves for serum glucose, insulin and amylin indicated a rise after the meal with peak concentrations at 30 min, as expected (*p* < 0.001; Appendix Figure A1). Both doses of SC lowered the peak serum glucose compared to placebo (*p* < 0.05, Appendix Figure A1a). *Salacia* had no significant effect on peak serum insulin or amylin concentrations, but both doses of SC lowered insulin and amylin at 60 min compared to placebo (*p* < 0.05; Appendix Figure A1b,c).

As expected, there was an increase in serum PYY and GLP-1, and a decrease in ghrelin after the meal (*p* < 0.001; Appendix Figure A1). Postprandial AUC for peak area for ghrelin (300 mg) was greater than placebo (*p* < 0.05), but did not reach significance at the 500 mg dose (*p* = 0.181) (Figure 4b). Serum PYY peak AUC was increased by both SC treatments compared to placebo (*p* < 0.05). GLP-1 had two peaks (60 and 120 min) after the meal that tended to be greater for SC than placebo (*p* < 0.06); however, SC did not significantly increase GLP-1 AUC compared to placebo (Figure 4b).

**Figure A2:**
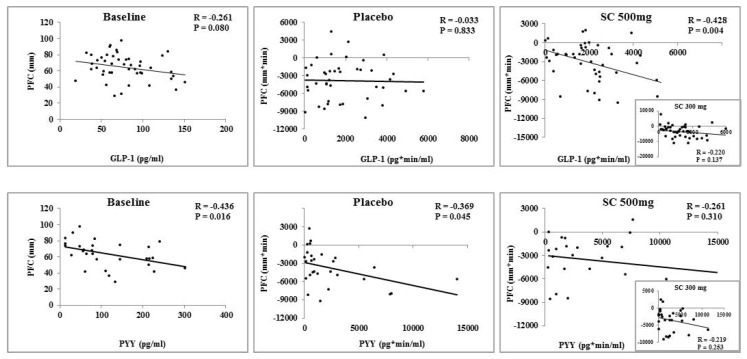
Correlations of gut hormones (GLP-1, PYY) and prospective food consumption (PFC) at baseline, and after the meal with placebo or SC treatment (SC 300 mg, SC 500 mg) using area under the curve.

There was an inverse relationship between baseline gut hormones (PYY and GLP-1) and appetite measures (hunger and desire for prospective food consumption) (*p* < 0.05; Appendix Figure A2). In addition, after the meal (placebo), PYY continued to be inversely correlated with a greater desire to eat (prospective food consumption) and positively correlated with fullness (*p* < 0.05; Appendix Figure A2). With SC treatment, only GLP-1 correlated with satiety and inversely with prospective food consumption (*p* < 0.05) (Appendix Figure A2).

### 3.3. Effect of Body Mass Index on Appetite and Serum Variables

In a separate analysis of the obese compared to overweight individuals, appetite measures were not significantly different at baseline (fasting). However, after the meal (180 min), the obese were hungrier, had a greater desire for prospective food intake and were less satiated (*p* < 0.02) with a trend for being less full (*p* = 0.06). Baseline serum glucose, insulin and amylin concentrations, and HOMA-IR were higher in the obese (*p* < 0.02), whereas QUICKI and the Matsuda index were lower in the obese than overweight (*p* < 0.001). Postprandial glucose, insulin and amylin total AUC was also greater in the obese than overweight (*p* < 0.001). With SC treatment, there were no significantly different effects in obese compared to overweight individuals for appetite measures, gut peptides or glycemic indices.

## 4. Discussion

The effect of α-GIs to decrease blood glucose and insulin levels and prevent the development of type 2 diabetes in patients with impaired glucose tolerance is well established [8,9,10,11,12,21]. There is limited evidence demonstrating an effect of α-GIs to enhance satiety [8,10], and none of them have reported its effect on both satiety and gastrointestinal hormones. The present study was designed to investigate whether the putative α-glucosidase inhibitor, *Salacia*, affects appetite and also to determine the role of gut peptides. We found no effect of SC on appetite in the entire population of healthy overweight/obese individuals, although there was a modest effect of SC on hunger in women. In addition, there were some greater postprandial increases in gut peptides with SC compared to placebo.

In the current study, SC displayed α-GI characteristics and decreased postprandial glucose and insulin, as expected [15]. In comparison to α-GI medications (i.e., mitglitol at 50 mg dose), that have been shown to reduce peak glucose by 14% and insulin by 55% at 60 min after a mixed meal test [8], SC decreased peak glucose by 6% and insulin by 11% at 60 min in the current study which may be partially attributed to our lower carbohydrate load. Amylin, which is co-secreted with insulin from pancreatic β-cells, increases in response to a glucose load [22,23]. In the present study, SC reduced postprandial amylin concentrations at 60 min, which is consistent with the reduced postprandial insulin and peak glucose concentrations.

In addition to their roles on glycemic control, animal studies and clinical trials indicate that α-GIs affect satiety and can promote weight loss [8,10,13,24]. In a study by Maggio et al., infusion of sucrose with acarbose in obese rats showed that this α-GI enhanced satiety [24]. In a randomized open-label crossover study in healthy volunteers, researchers examined the effect of miglitol on appetite. This study suggested that miglitol had an anorexigenic effect by increasing satiety and reducing hunger [8]. Moreover, in a double-blind clinical trial in obese patients with type 2 diabetes, miglitol significantly enhanced satiety, and reduced hunger [10]. Because glucose intolerance influences satiety [25], this may have contributed to the positive effects of miglitol on appetite [10]. In the present study, we recruited young healthy overweight/obese subjects who largely had normal fasting glucose and found no effect of SC on appetite measures in the entire group. However, as we largely recruited young healthy overweight/obese subjects, we were unable to test this hypothesis in the current study. A separate analysis in females indicated that there was less hunger after SC ingestion. It is possible that higher estrogen levels in females than males contribute to lower hunger feelings and the suppression with SC treatment [26,27,28]. It is also possible that, because men have higher feelings of hunger than women, as shown in the current study and by others [29], they are above a threshold whereby any effect on satiety becomes ineffective. Hence, the energy intake in the test meal that varies between studies, may affect the appetite responses to α-GIs and that various test meal loads should be considered [8,10]. Since other studies had used double the SC dose used here, a higher dose may be more appropriate in larger persons or in men; however, the higher dose was not effective in reducing hunger in women. It is possible that there is an ideal dose (not too high or low) needed to optimize the α-GIs properties vs. other properties of SC for a given meal size and this differs by sex.

Gastrointestinal hormones include peptides that are synthesized and released from the gastrointestinal (GI) tract and play essential roles in the normal regulation of GI function. It is well established that gut peptides could affect appetite and eating behaviors centrally and peripherally. In fact, gut hormone-based therapies are used in the treatment of appetite dysregulation disorders, such as obesity and anorexia-cachexia syndrome [5,30,31,32]. Importantly, it has been found that α-GIs enhance gut satiety signals. For example, miglitol administration in healthy participants significantly increases GLP-1 and PYY concentrations in healthy subjects or patients with type 2 diabetes [8,10]. In the current study in overweight and obese individuals, SC had modest effects on gut hormones. There was a significantly greater postprandial PYY peak area with SC than placebo, but the overall increase was small. In addition, postprandial GLP-1 only tended to be increased by SC compared to placebo. The smaller effect of SC on these anorexic peptides may be due to the relatively low carbohydrate load, and it is possible that the SC dose known to affect glycemic indices differs from the dose needed to maximally affect gut peptides. Ghrelin is an orexigenic gastro-intestinal peptide that is primarily released from the fundus of the stomach in response to hunger and signals the brain to increase food intake [33,34,35]. The effects of α-GIs on ghrelin are inconsistent in the literature. It has been reported that α-GIs significantly decrease postprandial circulating ghrelin in healthy subjects [8] and type 2 diabetic patients [36]. However, in another study of type 2 diabetic patients using a cross-over design, α-GIs, acarbose and miglitol showed no effect on postprandial ghrelin levels [37]. Moreover, in healthy men, the decrease in postprandial ghrelin concentrations was attenuated by the α-GIs, miglitol and vildagliptin or their combination, compared to placebo controls [9]. In another study with α-GI, acarbose, the suppression of ghrelin by oral sucrose was also attenuated by acarbose [38]. Similar results were found in the present study wherein the suppression of postprandial ghrelin was attenuated with SC compared to placebo. Since it has been found that the suppression of ghrelin is related with glucose load [39] and hyperinsulinemia [40], this may explain the attenuated postprandial ghrelin suppression by SC in the current study.

## 5. Strengths and Limitations

The primary purpose of this study was to examine the effect of SC on hunger and this is the first trial to examine this outcome as well as gastrointestinal hormones in a randomized controlled crossover design. Previous studies with *Salacia* have focused on glycemic indices due to its α-GI properties, indicating improved glycemic indices in healthy and diabetic subjects, and animals have been reported [15,16,19,41,42,43]. A similar effect was also observed in this study in overweight/obese individuals who were recruited to address our primary aim to examine appetite. In the current study, a small number of subjects had levels of fasting glucose >100 mg/dL, indicating prediabetes, but mean levels in this small group were near normal at 105 mg/dL. In addition, because we were not powered to examine whether there was a differential response to SC, creation of this small subgroup could not be addressed in this study. However, it would be interesting in a future study to also examine the glucose response of SC in patients with diabetes (or prediabetes) vs. healthy controls. Moreover, in the current study, we used a breakfast meal that had relatively low carbohydrate load compared to previous studies with α-GIs medication [8,9,10]. This can be considered a strength since SC lowered the glycemic response even in the presence of a low carbohydrate load that is both typical of a breakfast meal and more representative in patients adhering to a low carbohydrate diet. However, it is possible that without a larger meal and carbohydrate intake, their appetite remained above a threshold level whereby an α-GI may not effectively reduce hunger or affect gut peptides, thereby attenuating the response. In addition, this study was designed to examine the acute effect of SC on satiety after a fixed breakfast meal. This is because α-GIs act to reduce carbohydrate digestion and the rate of absorption at a given meal. However, it would be interesting to also examine repeated administration of SC in a future study since chronic intake of α-GIs may have differential effects compared to acute intake [44,45]. In addition, there may be other unknown active compounds in *Salacia* that have an appetite suppressant effect that would be more effective with chronic administration. Furthermore, in this study we used a visual analogue scale to measure appetite. An inherent limitation for measuring appetite with VAS is that there is wide variation between and within participants in this subjective measure that is dependent on individual′s feelings/standards. This limitation of the method has prompted those in the field to propose that VAS measurements with *p* values of less than 0.1 may be meaningful to avoid type II errors with these measurements [17]. However, to avoid increasing the risk of type I (false positive) errors, this higher *p* value was not used in the current study.

In conclusion, we found that *Salacia Chinensis* lowered glycemic indices in response to a meal. There was no overall effect of SC on appetite measures, but there was an effect on gut peptides, and hunger was attenuated in females. Future studies might consider whether these effects on gut peptides can affect appetite under different meal and dosing conditions or subsequent meal size to explain the modest weight loss effects observed with *Salacia* and α-GIs.

## Figures and Tables

**Table 1:** Characteristics of participants.

Age (year)	32 ± 12
Height (m)	1.69 ± 0.09
Weight (kg)	83.2 ± 15.5
Body mass index (kg/m^2^)	28.8 ± 3.6
Body fat (%)	27.4 ± 7.3
Waist circumference (cm)	98.8 ± 15.0
Blood pressure (systolic, mm Hg)	112 ± 13
Blood pressure (diastolic, mm Hg)	80 ± 8
Glucose (mg/dL)	85.9 ± 8.2
Insulin (µIU/mL)	16.8 ± 7.4
Amylin (pg/mL)	17.6 ± 10.8
GLP-1 (pg/mL)	78.4 ± 31.0
PYY * (pg/mL)	116.1 ± 81.0
Ghrelin (pg/mL)	375.9 ± 223.4

Values are means ± SD (range); *n* = 48 (* *n* = 33). Abbreviations: glucagon-like peptide-1 (GLP-1), peptide YY (PYY).
